# Severe pathological and transcriptional changes in haematopoietic organs of salmon suffering from salmon gill poxvirus disease

**DOI:** 10.1186/s12917-025-04922-6

**Published:** 2025-07-16

**Authors:** Ottavia Benedicenti, Ole Bendik Dale, Maria K. Dahle, Mona C. Gjessing

**Affiliations:** Norwegian Veterinary Institute, Postboks 64, Ås, 1431 Norway

**Keywords:** Salmon gill poxvirus, Erythrophagocytosis, Systemic response

## Abstract

**Background:**

Infection with salmon gill poxvirus (SGPV) can lead to acute disease outbreaks with high mortalities caused by extensive gill pathology. In some cases, additional signs of severe red blood cell breakdown (erythrophagocytosis) are present in the spleen and kidney. This can indicate a broader systemic effect of the infection, extending beyond the gills. In a previous study, we investigated the gill histopathology and transcriptome response of salmon sampled from such an outbreak. Here, we further investigate these responses in the same fish, focusing on the haematopoietic organs.

**Results:**

Results show that extensive accumulation of red blood cell breakdown products and phagocytosis of red blood cells were seen in the salmon suffering from salmon gill poxvirus disease. Moreover, differentially expressed genes exhibited an apparent organ-specific pattern, with a primary function involved in immune response, which was predominantly observed in the spleen and kidney. Additional antiviral responses, as well as coagulation and vascular function, apoptosis, and stress responses, were also detected in haematopoietic organs.

**Conclusions:**

In conclusion, salmon gill poxvirus disease (SGPVD) affects haematopoietic organs, causing red blood cell breakdown and organ-specific immune responses. Gene expression patterns highlight immune activation, antiviral defence, coagulation, and stress pathways in the spleen and kidney of salmon suffering from SGPVD.

**Supplementary Information:**

The online version contains supplementary material available at 10.1186/s12917-025-04922-6.

## Background

The salmon gill poxvirus disease (SGPVD) has emerged in Atlantic salmon (*Salmo salar* L.) aquaculture in the last couple of decades. Severe SGPVD in hatcheries is often seen as outbreaks confined to tanks that have strikingly synchronised onset and a disease course lasting for a few days, with very high mortalities. These “tank outbreaks” can manifest as a pestilence, randomly spreading to new tanks, but with the same strikingly synchronised occurrence. Infection with salmon gill poxvirus (SGPV) in fish at sea is sometimes one of the players in complex gill disease [1]. SGPV was first identified in Norway in the 1990s, and its large DNA genome was partly characterised in 2015 [2–4]. The virus has also been detected in the Faroe Islands, Scotland, Iceland and Canada [4–9]. Infection with SGPV can cause acute respiratory disease, leading to high mortality in Atlantic salmon pre-smolts, but in other cases to no clinical disease at all. While gill pathology has been investigated in several studies, systemic features, such as erythrophagocytosis in the spleen and kidney, are less understood. In salmon suffering from SGPVD, characteristic histopathological changes are seen in the gills with a large part of the gill respiratory surface being impacted. Apoptosis of gill epithelial cells containing SGPV particles, as confirmed by terminal deoxynucleotidyl transferase dUTP nick end labelling (TUNEL)-positive staining, transmission electron microscopy and RNAscope in-situ hybridisation, is a hallmark of SGPVD. The degree of apoptosis is quantitatively linked to SGPV load and disease severity [9, 10]. In some cases of severe SGPVD, extensive changes are observed in the spleen and kidney, including erythrophagocytosis - the phagocytic destruction of red blood cells (RBCs) [4, 9, 10]. The outcome of SGPV infection is highly unpredictable and the process leading to acute, synchronised disease outbreaks and high mortalities is still poorly understood. Understanding the true nature of these dramatic disease manifestations is vital for improving management strategies. In addition to severe histopathological changes in the gill and haematopoietic organs, there are, in some cases, fish with sharply delineated orange-reddish discolouration of skin / fins, muscular tetanus (dubbed “live rigour”) [11], alterations in blood appearance and consistency, and sometimes necrosis of entire gill filaments. This pathology and acute mortality could be attributed to microvascular thrombotic disorders resulting from consumptive coagulopathies, often associated with septicaemia, also in fish. However, conclusive evidence of SGPV replication outside the gill epithelium remains elusive, making it difficult to ascribe the pathology of the spleen and kidney to septicaemia.

The Atlantic salmon transcriptional response in gills during a natural outbreak of SGPV showed an increased expression of pro-apoptotic genes in line with the pathological findings, as well as changes in ion channels and mucins [10]. Moreover, the innate antiviral response was strongly up-regulated, while mucosal defence mechanisms were suppressed [10]. In an experimental study, fish injected with hydrocortisone before SGPV infection exhibited more severe gill pathology and higher viral loads [12, 13]. The transcriptional response to hydrocortisone-injected fish included delayed expression of genes such as myxovirus resistance (*Mx*), interferon-gamma (*IFNγ*), and granzyme A (*graa*), which are involved in antiviral defence and cytotoxic cell activity [12]. Moreover, the systemic immune response was also induced in the spleen with the up-regulation of *IFNγ* and *graa* [12]. The spleen is a key lymphoid organ in fish and is involved in immune function and blood filtration and consists of red pulp, rich in RBCs, and white pulp, containing lymphocytes for immune response [14]. The teleost kidney is a multifunctional organ divided into the head, mid, and posterior kidney, with the head kidney being the major haematopoietic and endocrine organ, while the posterior kidney has nephrons for osmoregulation and urine production. The mid-kidney acts as a transition zone between these two parts [15]. Moreover, the kidney and spleen are the main organs for phagocytic activity in teleost [16–18].

In this study, we conducted a further investigation on fish from a natural outbreak of SGPVD, the same individuals which had previously been examined with a primary focus on the gills [10]. To gain a more comprehensive understanding of the disease processes occurring in the haematopoietic organs (kidney and spleen), we investigated these organs using a combination of histology and transcriptomics.

## Results

### Clinical manifestation of the SGPVD outbreak

The disease course of this outbreak has also been described earlier by Gjessing et al. 2020. Briefly, the SGPVD outbreak occurred a few days after sorting in the spring of 2017 and lasted from May 10^th^ to June 4^th^, with more than 200,000 Atlantic salmon pre-smolts dying with clinical signs typical of SGPV infection. In the mortality tanks, the fish had been sorted some days before the outbreak. Kidney and spleen from the same animals where gills had been investigated previously [10], were included in this study (Table 1). From tank I (M_I group), samples were collected from five diseased fish upon the onset of clinical disease, coinciding with the increasing mortality, then sampled again eight days after, when the fish no longer showed behavioural signs of disease (Fig. S1). Four clinically healthy fish, identified as the L_I group, were sampled to monitor the late phase of the infection (Fig. S1). Diseased fish in tank II were observed eleven days after the onset of the disease in tank I, and samples were collected from five clinically diseased fish, identified as the M_II group, two days after the disease onset and at the peak of mortality (Fig. S1). Tank III served as a control tank in this study, and six and four fish were sampled concurrently with the outbreaks in tanks I and II, respectively. No clinical signs of disease were observed during sampling in tank III. However, a sudden increase in mortality was reported after eight days. The initial sampling from tank III was utilised as a negative control sample and labelled as the C_III group (Fig. S1). The gills from the second sampling were found to be SGPV positive [10], considered early pre-outbreak samples, and denoted as the E_III group (Fig. S1).

### Histopathological findings

**Fig. 1:**
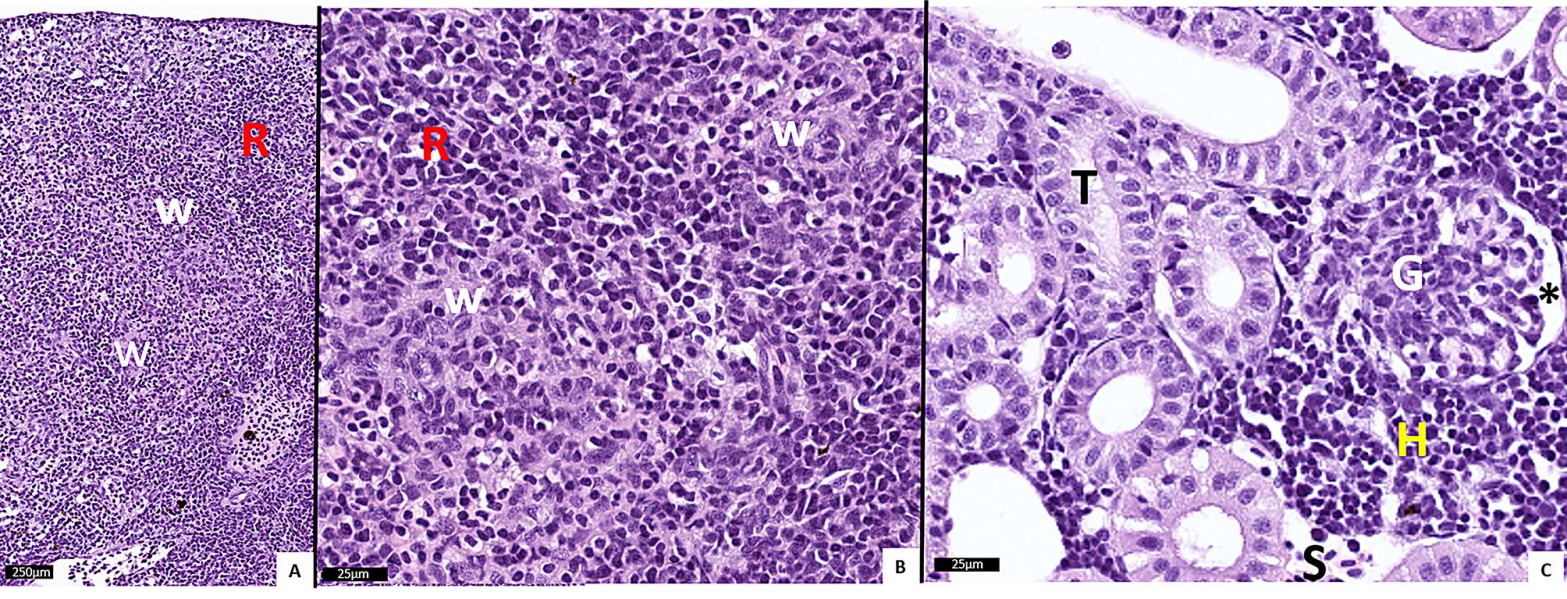
H&E-stained histological sections of clinically normal spleen (**A**-**B**) and kidney (**C**) from salmon in the C_III group. **A**-**B**: White pulp indicated by W, red pulp indicated by R. **C:** Normal tubuli (T), glomerulus (G, asterisk in Bowmans room), haematopoietic tissue (H), sinusoid (S).

In all fish analysed, vacuolization of hepatocytes and only very minor and unspecific lesions were seen in the heart, skin, pancreas / pyloric caeca. In the gills (C_III and E_III groups) and spleen (Fig. 1A-B) and kidney (Fig. 1C) of the C_III group, no or only very minor histopathological lesions were seen.

In the E_III group, 10 days before the disease outbreak in that tank, sparse circulatory disturbances were seen in the spleen and kidney. In addition to the previously reported severe SGPV gill infection and extensive apoptosis of gill epithelial cells in the M_I and M_II group [10], striking histopathological changes were present in the spleen and kidney in these fish.

**Fig. 2:**
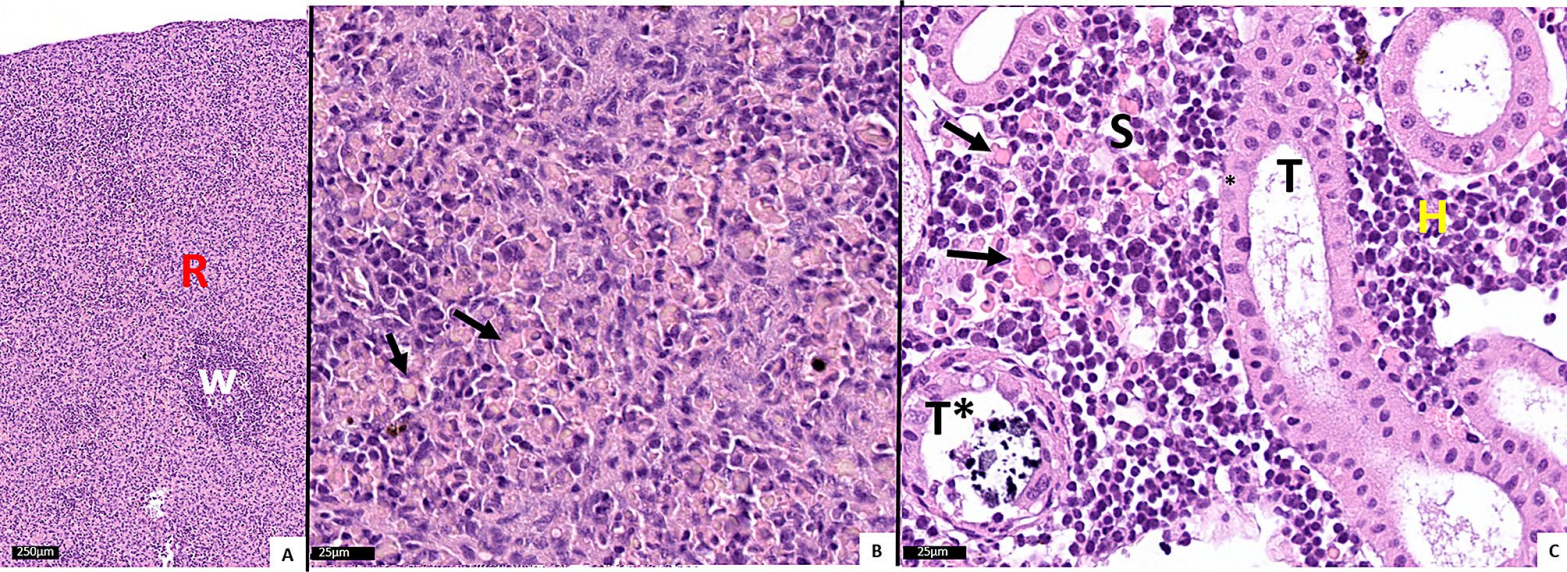
H&E-stained histological sections of spleen (**A**-**B**) and kidney (**C**) from the M_I group. **A**: Note the prominent distinction between red (R) and white (W) pulp. **B**: magnified area from red pulp of A, with arrows pointing at phagocytosed RBCs. **C**: Extensive haemophagocytosis seen in sinusoids (S) and indicated by arrows. H indicates area of haematopoietic tissue, T and T* indicate tubulus and tubulus with calcified deposits.

**Fig. 3:**
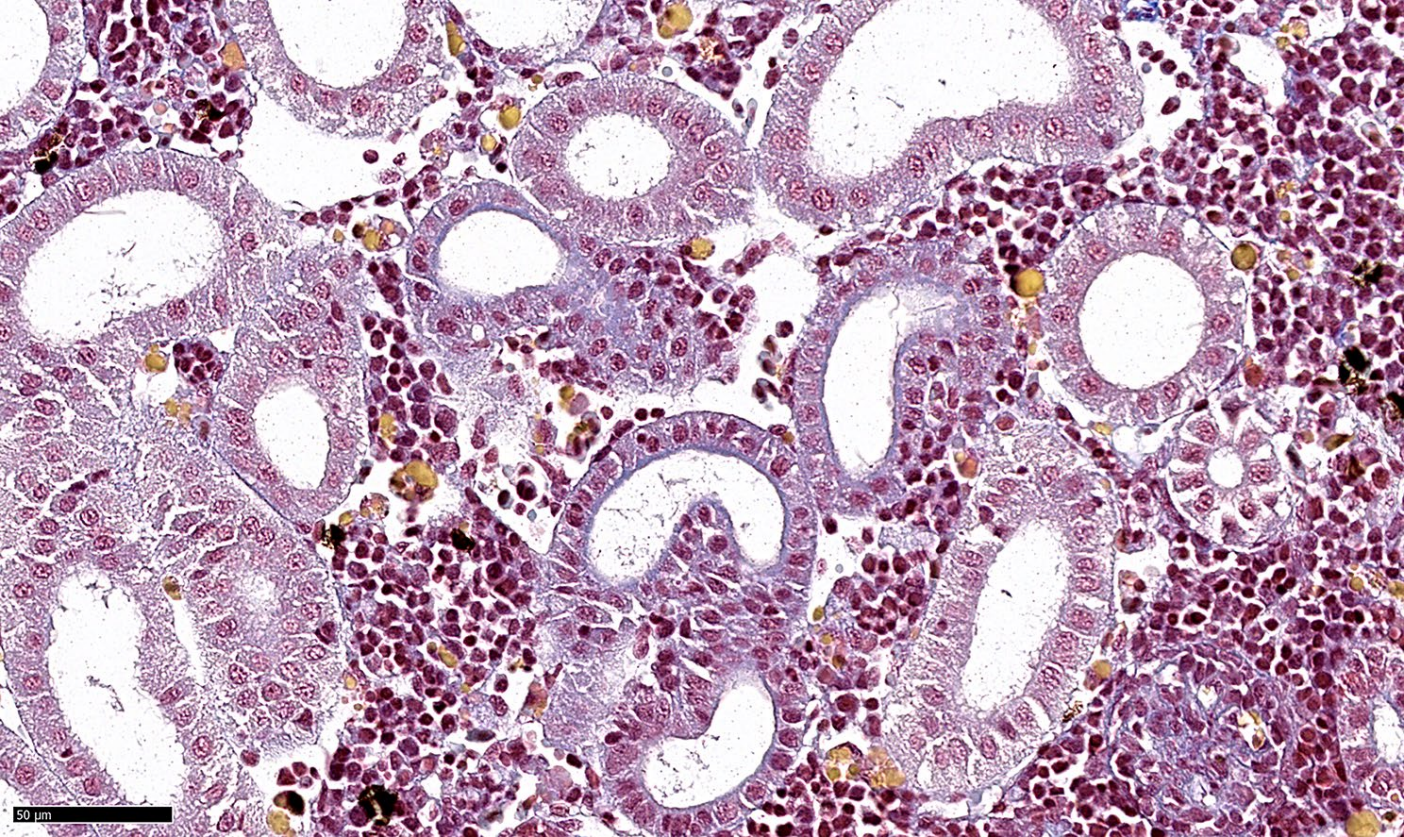
MSB-stained histological sections of the kidney from the M_I group. Yellow staining (same staining properties as RBCs) of the deposits seen in Fig. 2C suggests that these are phagocytosed RBCs.

In the spleen, normal red pulp was difficult to find, as severely increased amounts of amorphous (hyaline) eosinophilic deposits were seen in this compartment (Fig. 2A). Such deposits, suspected to be extensive erythrophagocytosis (i.e., phagocytosed RBCs) (Fig. 2B), were also seen in the kidney, lining or inside sinusoids (Fig. 2C). Moreover, the Martius, Scarlet and Blue (MSB) staining method was used to confirm the yellow staining of deposits, suspected to be phagocytosed RBCs, and also to investigate the presence of fibrin (Fig. 3).

**Fig. 4:**
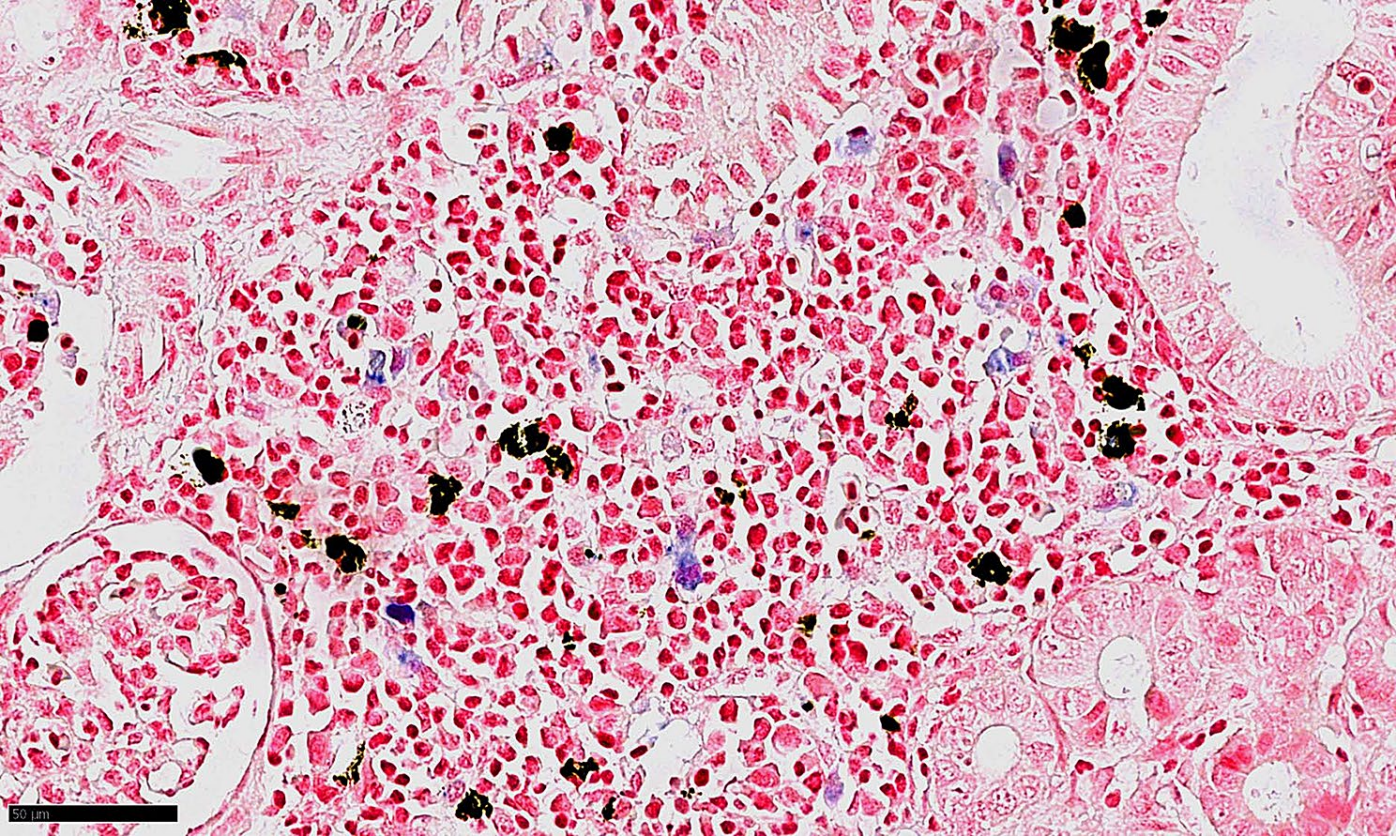
Prussian blue-stained histological section of the kidney of fish from the L_I group.

In the L_I group, sparse lesions were seen in the gills as reported previously [10] and also in the spleen and kidney. As the iron in phagocytosed RBCs will become oxidised over time and aggregate into hemosiderin, we performed a Prussian blue stain on a subset of spleen and kidney sections from the L_I group. The method detects the ferric (Fe³⁺) iron in hemosiderin, forming an insoluble blue pigment and some staining of hemosiderin-like deposits was seen in the L_I group (Fig. 4).

### Transcriptomic analyses

**Fig. 5:**
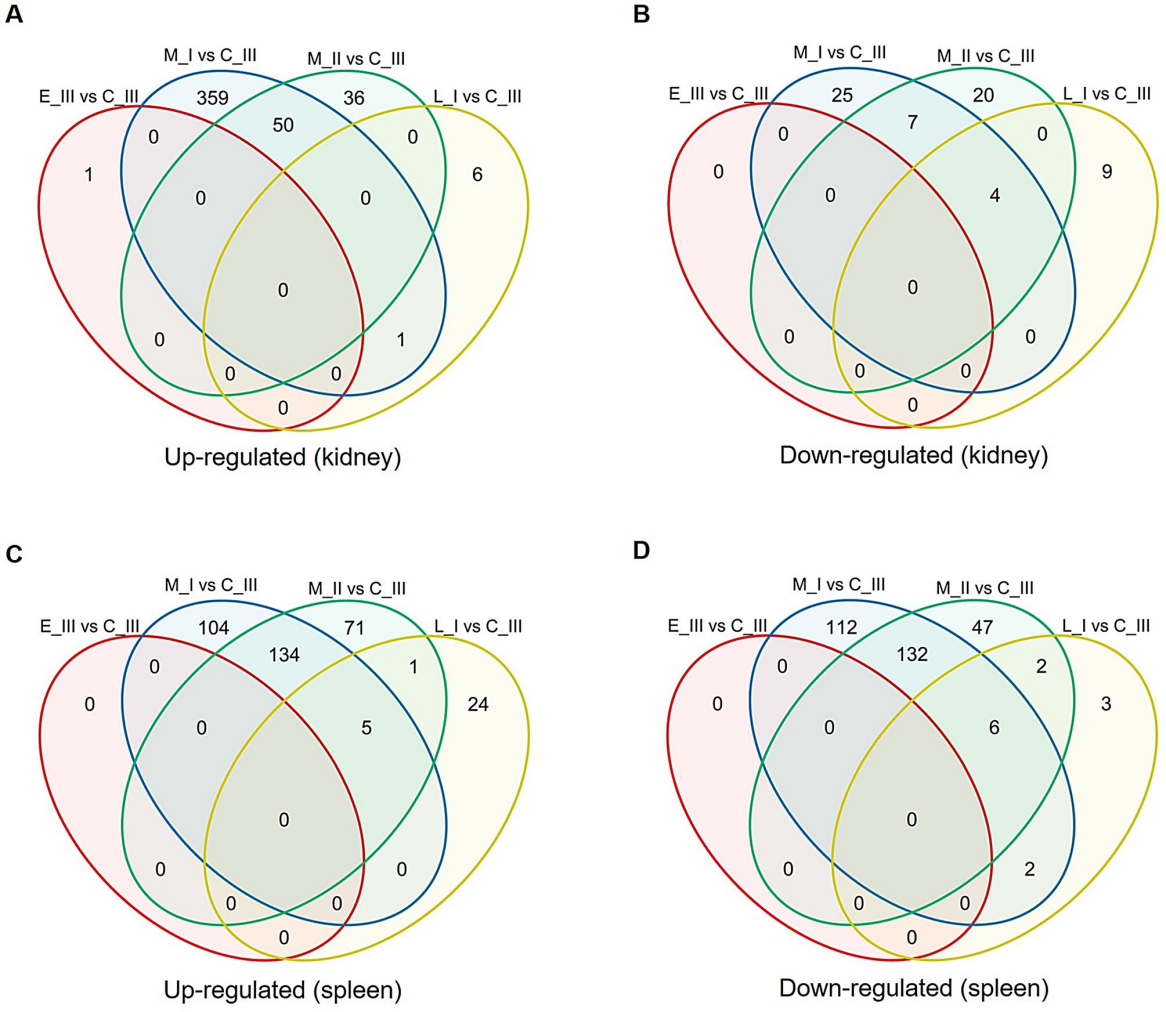
Venn diagrams illustrating the number of shared and unique DEGs across all sampling groups for each tissue. Panels **A** and **B** represent kidney samples, while panels **C** and **D** correspond to spleen samples. DEGs were identified using a threshold of|log₂ fold change| ≥ 1 and an adjusted p-value ≤ 0.05.

Twenty-four representative samples from all sample groups (Table 1) were selected for transcriptomic analyses on the kidney and spleen. The animals that were selected included the same fish where the disease processes and transcriptome of gills were previously investigated, and where extensive gill pathology with high gill SGPV load was found [10]. Here, overall, the kidney and spleen transcriptional profiles showed considerable variation in the principal component analysis (PCA), revealing that two clusters were formed per organ, each of them with considerable overlap among sampling points (Fig. S2A). Moreover, PCA multiple small barplot analysis illustrated the distribution of variables along the first four principal components (Fig. S2B). To understand the range of transcriptional responses to SGPV infection better, we performed a comparison of infected (M_I, L_I, M_II, and E_III) and uninfected fish (C_III) for each organ (Table S1), taking into account that C_III fish group was sampled at the first sampling point (Table 1). Our results showed that genes are mainly differentially expressed when comparing M-I and C-III, or M-II and C-III for both organs (M_I vs. C_III: *n* = 410/up-regulated and 36/down-regulated in kidney and *n* = 243/up-regulated and 252/down-regulated in spleen; M_II vs. C_III: *n* = 86/up-regulated and 31/down-regulated in kidney and *n* = 211/up-regulated and 187/down-regulated in spleen), while only a few genes were found differentially expressed in the post-disease outbreak fish (L_I vs. C_III, *n* = 7/ up-regulated and 13/down-regulated in kidney and *n* = 30/up-regulated and 13/down-regulated in spleen) (Fig. 5). Overall, spleen responses were dominated by immune genes, while kidney responses included immune, apoptotic, and vascular gene regulation, mostly during acute infection.

**Fig. 6:**
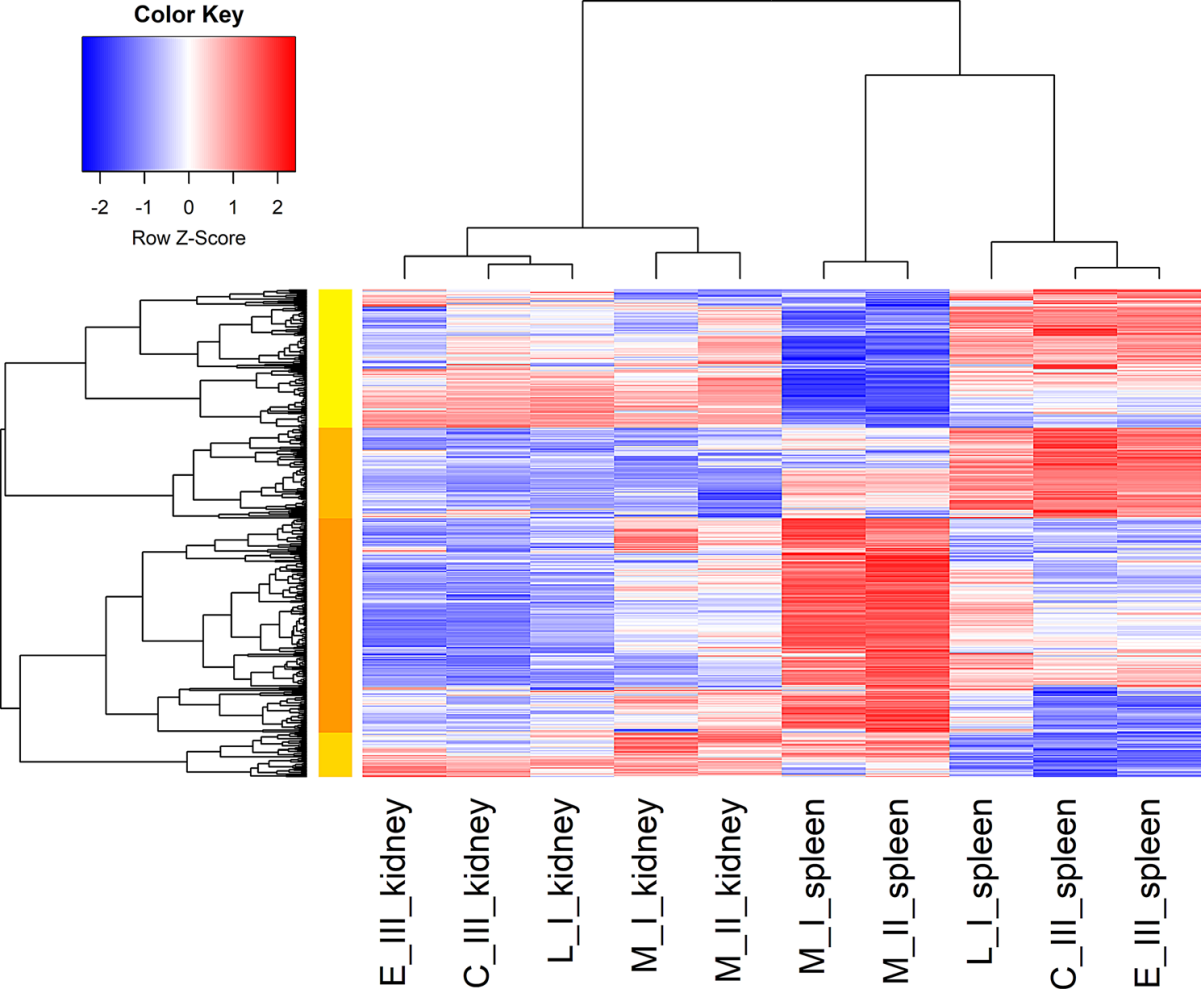
Heatmaps displaying DEGs in the spleen and kidney. Expression values are represented as row Z-scores, which standardise gene expression across samples to highlight clusters of genes with similar expression trends. This visualisation enhances the comparison of gene regulation patterns between tissues and sampling groups.

A heatmap based on the infection-related differentially expressed genes (DEGs) (M_I vs. C_III spleen contrast) illustrated this difference in expression patterns between the two organs (Fig. 6). Broadly when considering only genes that were differentially expressed during the sampling points of diseased fish (M_I and M_II), these two groups clustered together for each organ (Fig. 6). Moreover, the control genes’ profile is more closely related to the recovered fish for kidney, while control and early infection groups cluster together for spleen (Fig. 6). This latter finding was also shown in the Venn diagrams, where spleen showed more up-regulated DEGs in surviving fish vs. control fish compared to kidney (L_I vs. C_III) (Fig. 5).

**Fig. 7:**
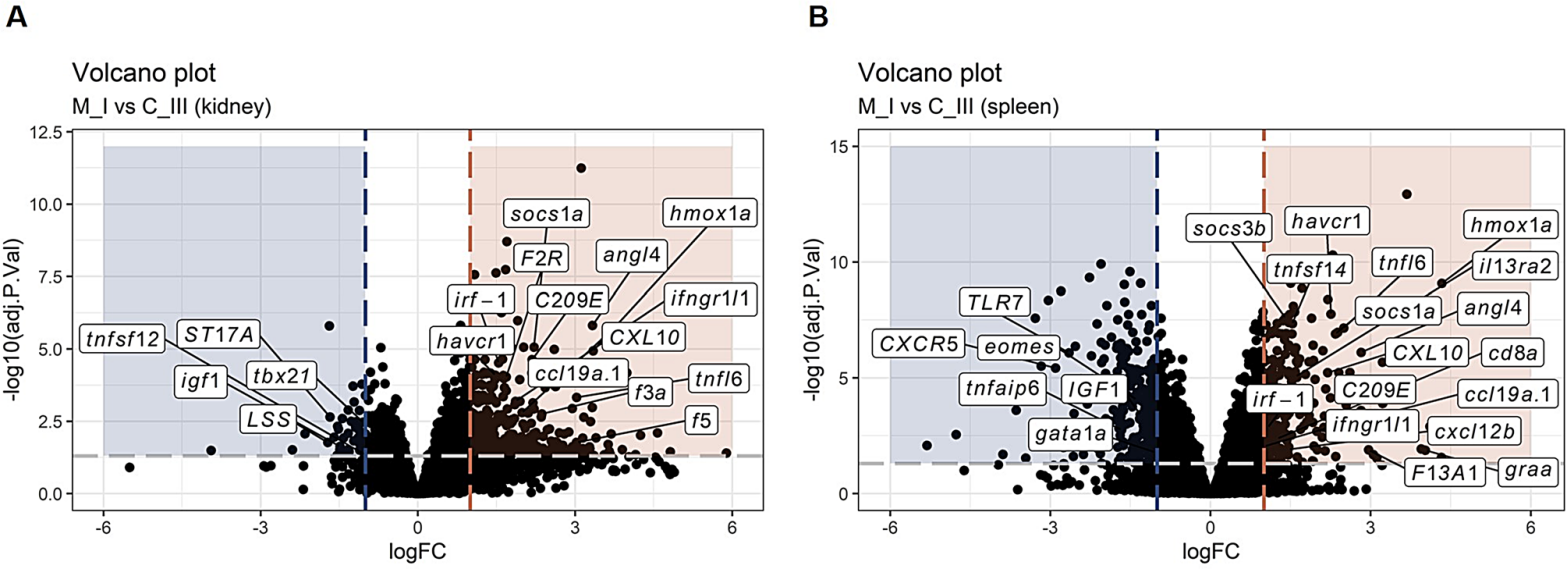
Transcriptional response in kidney and spleen (M_I vs. C_III). Volcano plot in kidney (**A**) and spleen (**B**) samples showing DEGs (log_2_ fold change of │1│ and adjusted p-value ≤ 0.05).

**Fig. 8:**
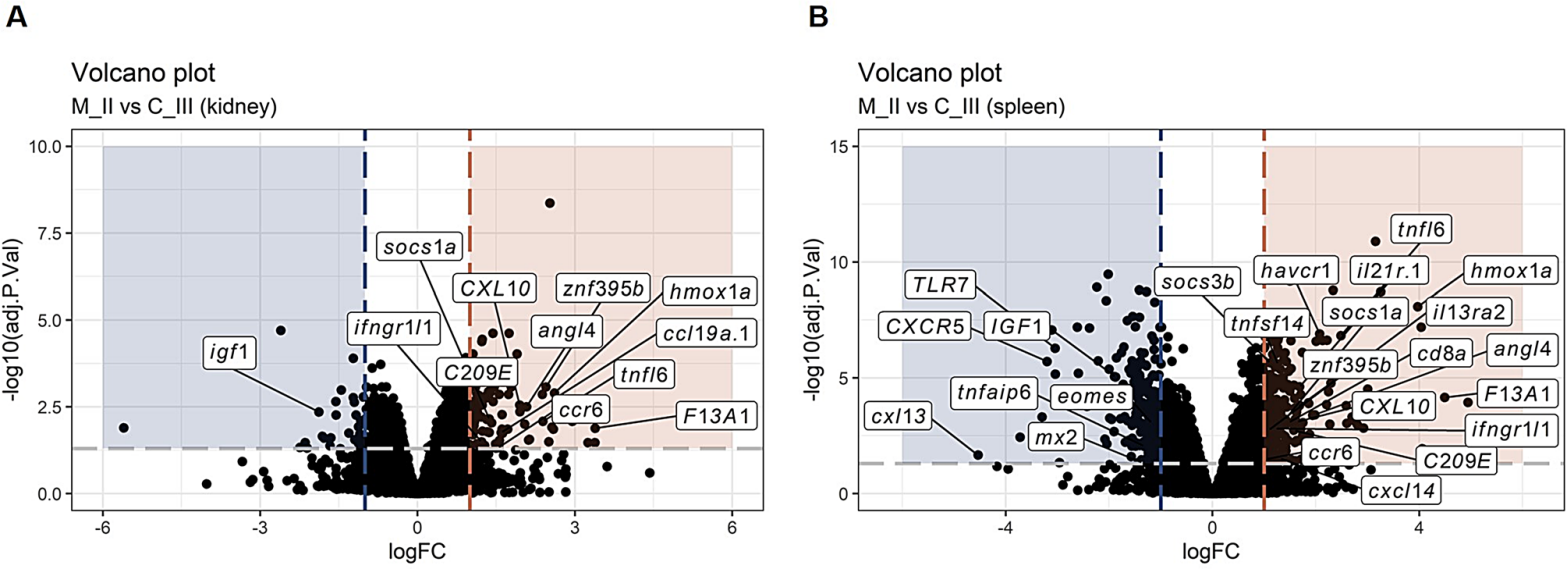
Transcriptional response in kidney and spleen (M_II vs. C_III). Volcano plot in kidney (**A**) and spleen (**B**) samples showing DEGs (log_2_ fold change of │1│ and adjusted p-value ≤ 0.05).

**Fig. 9:**
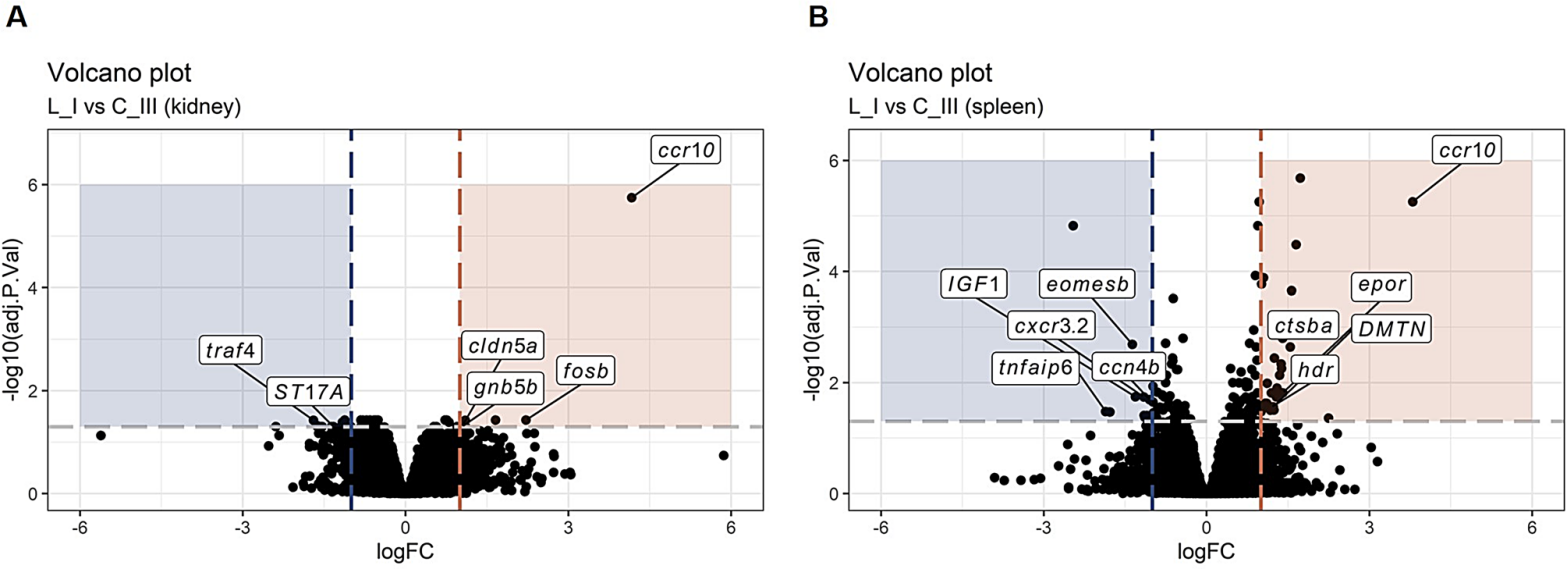
Transcriptional response in kidney and spleen (L_I vs. C_III). Volcano plot in kidney (**A**) and spleen (**B**) samples showing DEGs (log_2_ fold change of │1│ and adjusted p-value ≤ 0.05).

DEGs were further investigated based on their putative function, as most of the genes appeared to be involved in immune response, coagulation / vascular function, antiviral response, apoptosis-related response or stress / hormone response (Figs. 7, 8 and 9).

For the M-I and M_II vs. C_III comparison, the immune response-related genes dominated more in the spleen vs. a mix of antiviral / apoptosis / immune-related responses in the kidney (Figs. 7 and 8). Moreover, in the M_II vs. C_III and L_I vs. C_III comparisons, most DEGs were mainly present in the spleen compared to the kidney (Figs. 8 and 9). *socs1a*, also known as suppressor of cytokine signalling (SOCS), is a cytokine-inducible negative regulator of cytokine signalling. Induction of this gene is commonly associated with an immune response, and it was found up-regulated in both organs in the disease groups of this study (M_I and M_II), and in gills from the same fish as previously described [10] (Figs. 7 and 8). Other up-regulated genes putatively involved in an immune response and shared between the two organs were the tumour necrosis factor ligand superfamily member 6 (*tnfl6*) [10, 19] and the heme oxygenase 1a (*hmox1a*) [20] (Figs. 7 and 8). The tumour necrosis factor ligand superfamily member 6 is part of the FAS/FASLG signalling pathway, which is essential for immune system regulation, including activation-induced cell death (AICD) of T cells and cytotoxic T lymphocyte-induced cell death. Moreover, *tnfl6* is involved in induced erythropoiesis during acute anaemia in Atlantic salmon [21]. The heme oxygenase gene plays a role in several biological processes and is also responsive to hypoxia stress [22]. *hmox1a* has also been found to be associated with a cardiac response to stress [23]. Concerning a vascular function, the angiopoietin-related protein 4 (*angl4*) [24] was found up-regulated in both organs during the disease (M_I and M_II vs. C_III) (Figs. 7 and 8), while most of the coagulation factors (*F2R*, *f3a*, *f5*) were found up-regulated mainly in the kidney during the disease sampling in tank I (Fig. 7A).

The C-X-C motif chemokine 10 (*CXL10*) [25], the C-type lectin domain containing 1 (*C209E*) [26, 27] and interferon-gamma receptor 1-like 1 (*ifngr1l1*) [28] are usually associated to an antiviral response, and they were found up-regulated in both organs during the disease (M_I and M_II vs. C_III) (Figs. 7 and 8).

Most of the DEGs putatively associated with apoptosis or stress/ hormone response were mainly found up-regulated in the kidney compared to the spleen during the disease sampling in tank I (Fig. 7A) and in the spleen compared to the kidney during the disease sampling in tank II (Fig. 8B). The insulin-like growth factor 1 (*igf1*) is involved in mediating growth and development [29] and it was found down-regulated in both organs during the disease (M_I and M_II vs. C_III) (Figs. 7 and 8). As previously found by Amundsen et al. (2021), the cluster of differentiation 8a (*cd8a*), found on most cytotoxic T lymphocytes, and the *graa*, a T cell-specific serine protease that may function as a common component necessary for lysis of target cells by cytotoxic T lymphocytes, were found up-regulated in the spleen during the disease phases (Figs. 7B and 8B).

Only a few genes were found differentially expressed in kidney in surviving fish (L_I vs. C_III) (Fig. 9A). However, the chemokine (C-C motif) receptor 10 (*ccr10: ssCXCR6/drCCR10*) [30] was found up-regulated in both organs in surviving fish (Fig. 9). CCR10 is reported with a main role to direct T-cells to skin in mammals [31]. Moreover, also up-regulated in survivors were genes putatively associated with the coagulation / vascular function in the spleen: erythropoietin receptor (*epor*) with a role in erythroid cell differentiation and survival [32], the dematin actin-binding protein (*DMTN*), which plays a structural role in erythrocytes [33], and the haematopoietic death receptor (*hdr*), which may function as a negative regulator of erythropoiesis [34].

**Fig. 10:**
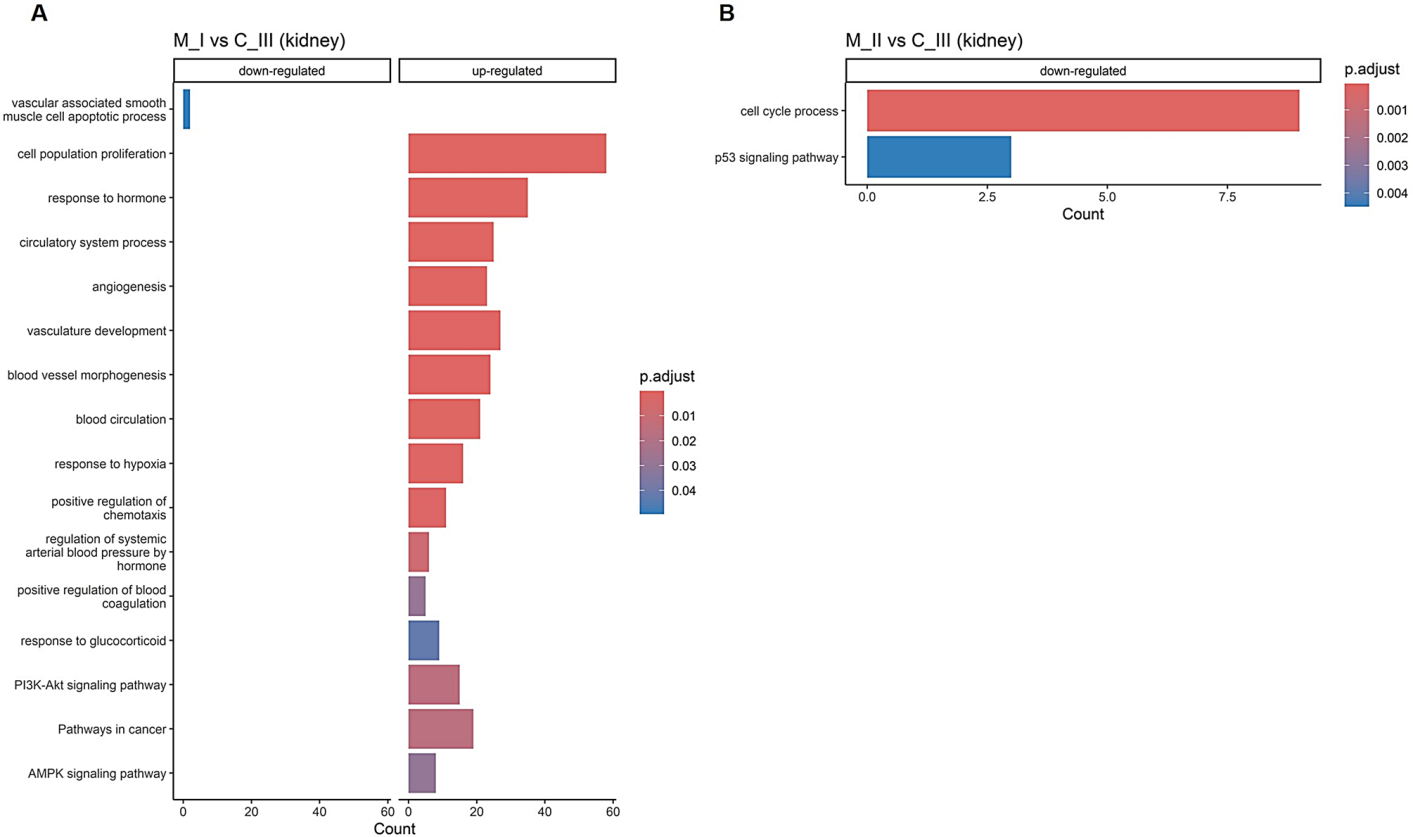
Bar charts showing the enriched gene ontology in up- and down-regulated genes in the kidney. (**A**) shows M_I vs. C_III and (**B**) shows M_II vs. C_III. The count axis refers to the number of genes (or other annotated entities) associated with a specific GO term.

**Fig. 11:**
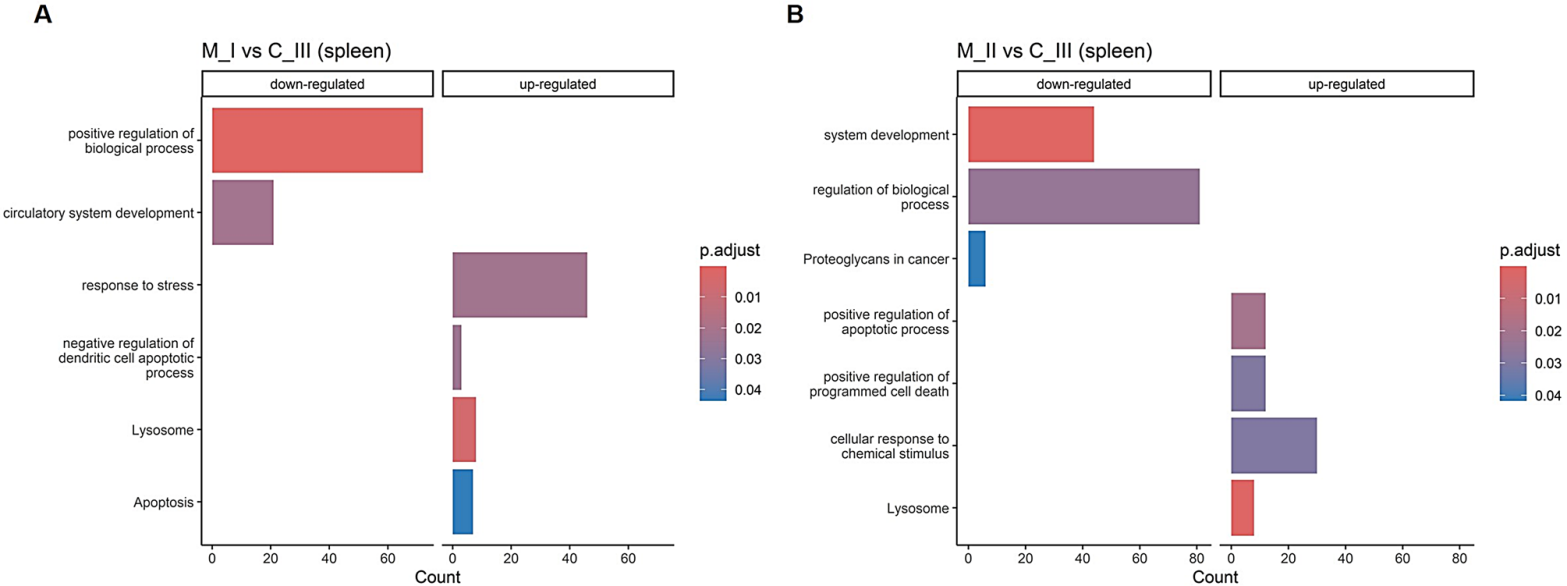
Bar charts showing the enriched gene ontology in up- and down-regulated genes in the spleen. (**A**) shows M_I vs. C_III and (**B**) shows M_II vs. C_III. The count axis refers to the number of genes (or other annotated entities) associated with a specific GO term.

Gene ontology (GO) enrichment analysis of the organ-specific DEGs in each of the diseased fish groups (M_I and M_II vs. C_III) revealed two different enrichment profiles in the two organs (Figs. 10 and 11). In the kidney, most of the GO biological processes were characterized in the M_I vs. C_III fish, with the involvement of up-regulated genes associated with coagulation / vasculature signatures (i.e., angiogenesis, blood circulation, positive regulation of blood coagulation, vasculature development, and blood vessel morphogenesis), chemotaxis and response to stress / hypoxia (Fig. 10A). In the kidney M_II vs. C_III comparison, only the p53 signalling pathway indicated increased cell apoptosis (Fig. 10B) [35]. GO enrichment analysis in the spleen showed mainly up-regulated genes associated with apoptosis processes and the lysosome KEGG pathway, indicating the involvement of potential mechanisms of phagocytosis / autophagy (Fig. 11) [36].

## Discussion

This SGPVD outbreak and these fish have previously been investigated and described with the gill pathology and responses as the main focus [10]. Here, we further investigate these fish through an interdisciplinary approach, adding another piece to the puzzle. We studied the haematopoietic organ histopathology and transcriptomic responses. Extensive erythrophagocytosis was seen in the mortality phase, and differentially expressed genes were mainly seen in diseased fish with apparently organ-specific patterns. Their main function was involved in immune response, but also antiviral response, coagulation and vascular function, apoptosis and stress responses were seen.

Erythrophagocytosis is a normal physiological process, occurring primarily in the spleen and kidney in salmon, where old or damaged erythrocytes are continuously removed by macrophages [37]. Infections, trauma or oxidative stress are examples of conditions that can lead to increased breakdown of RBCs in teleost [38, 39]. An example is infectious salmon anaemia virus, a virus that replicates in endothelial cells, but that can adhere to RBCs and lead to increased erythrophagocytosis causing anaemia, one of the hallmarks of infectious salmon anaemia [39]. Under these conditions, the RBCs-breakdown may result directly from the interaction between the virus and RBCs. Previous studies of salmon affected by SGPVD did not detect any evidence of SGPV infection within RBCs at the time of sampling (unpublished). Therefore, the underlying mechanisms of erythrophagocytosis in these two diseases are likely to differ. In fact, the severity and acuteness of erythrophagocytosis of the spleen and kidney of the salmon seen in this study are striking and have not been previously reported to this extent in other specific infectious salmon diseases. The kidney sinusoids are lined by endothelial cells and macrophages [40]. The endothelial cells in the salmon kidney are specialised for the uptake of soluble macromolecules via receptor-mediated endocytosis, rather than for the phagocytosis of entire cells such as RBCs. Therefore, the observed erythrophagocytosis in the kidney is likely mediated by macrophages residing within the sinusoid lumen. Oxidative stress, which is anticipated to play a role in SGPVD, may accelerate RBCs-breakdown by damaging their membranes and altering surface markers, thereby facilitating erythrophagocytosis. In the pancreas disease (PD), a viral disease of salmon, we see endothelial scavenger cells filled with small granules, presumably consisting of myoglobin from severe muscle necrosis [41]. These granules have the same staining characteristics as in SGPV and infectious salmon anaemia virus (ISAV). At the molecular level, *igf1* expression stimulates cell growth and inhibits apoptosis, and it has been shown that disruption of *igf1* signalling can lead to oxidative stress [42, 43], and *hmox1a* is involved in the protection of multiple tissues and organs from oxidative stress and excessive inflammatory reactions [44]. The down-regulation of *igf1* and the up-regulation of *hmox1a* suggest a role for oxidative stress in RBCs-breakdown.

Many genes related to coagulation were found up-regulated during acute disease in the kidney, while genes associated with erythroid cell functions, including maturation and survival [32], structure of erythrocytes [33], and inhibition of erythropoiesis [34] were found up-regulated in the spleen of post-disease outbreak fish. Moreover, *tnfl6* known to induce erythropoiesis during acute anaemia in Atlantic salmon [21], *angl4* involved in the vascular function [24], and most of the coagulation factors (*F2R*, *f3a*, *f5*) were found up-regulated in diseased fish. The interplay between viral infections and coagulation processes in fish is still not well understood. Certain viruses can induce haemorrhagic conditions that affect blood clotting mechanisms in fish (viral haemorrhagic septicaemia virus-VHSV, infectious haematopoietic necrosis virus-IHNV [45], and ISAV [46]). However, detailed mechanisms of how these viruses affect the coagulation pathways in fish remain unclear.

The transcriptomic response in haematopoietic organs of fish suffering from SGPVD compared to healthy controls of this study revealed that most of the DEGs were found in the acute disease phase of the fish, just a few in the post-disease outbreak fish, and almost none in the early infection stage. This contrasted with what was found for the transcriptomic response in the gills of these fish, with some DEGs also during early infection [10], probably caused by early SGPV infection in the gills. An organ-specific gene expression pattern is expected in the gills, kidney and spleen, reflecting their distinct physiological roles, cellular composition, and responses during an infection. In the gills of these fish, a complex transcriptomic response, with marked activation of antiviral pathway genes during the acute phase of SGPV infection, modulation of gill epithelial cell apoptosis and cell proliferation genes in line with gill hyperplastic changes, suppression of genes involved in mucosal defences, and a delayed adaptive immune response were seen [10]. These changes collectively aligned with the observed gill pathology, the clinical manifestations of SGPVD [10] and the cellular composition of gills (respiratory epithelial cells, mitochondria-rich cells, mucous cells, immune cells, and endothelial cells). The gene expression pattern reflected the spleen’s simpler cellular composition, with immune response-related genes predominating during the acute phase. In contrast, the kidney exhibited a more complex expression profile, with a mix of antiviral, apoptotic, and immune-related responses. The CD8α protein is a part of the CD8 co-receptor found on the surface of certain immune cells, particularly cytotoxic T lymphocytes or CD8^+^ T cells. Upon recognition of an infected or abnormal cell, CD8^+^ T cells use their CD8 co-receptor to trigger the release of cytotoxic substances (like granzyme) to kill the target cell. The *cd8a* and *graa* genes were found both up-regulated in the gills [10] and spleen in the M_I and M_II groups in this study, a response that is delayed, but more pronounced in fish pre-treated with hydrocortisone, correlating with increased gill pathology and mortality [12]. These findings suggest that SGPVD trigger a cytotoxic immune response during the acute phase of the infection, aimed at controlling infection severity, although the timing and intensity of this response could be influenced negatively by factors such as stress-induced immunomodulation. SOCS1 negatively regulates interferon signalling, which can reduce the ability of the body to clear viral infections. It is a protein that plays a critical role in regulating the immune system and controlling inflammatory responses. It does so by inhibiting various signalling pathways and acts as a negative feedback regulator to prevent excessive immune responses that could lead to tissue damage. In this study, *socs1a* was found up-regulated in both organs during the disease (M_I and M_II) and was also up-regulated in the gills in these animals [10], and it might contribute to the suppression of mucosal defence mechanisms in the gills, potentially facilitating viral persistence and progression of the disease. However, in mice, *socs1a* protects from lethal infection with another poxvirus, the vaccinia virus [47]. In fish, the potential of specific gene expression changes, such as *socs1a*, to serve as an early biomarker for disease detection or monitoring warrants further investigation and could lead to valuable future clinical applications. Moreover, in mammals, there is evidence suggesting that the host immune response to poxvirus infections can lead to an overproduction of cytokines, causing more harm than the infection itself, resulting in a “cytokine storm” that contributes to disease pathology [48, 49]. *socs1* also serves as a modulator of macrophage function and, while its direct role in haemophagocytosis is not established, its influence on macrophage activity suggests that alterations in its expression could impact RBCs turnover [50].

In the cases of rapid, high mortality, we see widespread apoptosis of SGPV-infected gill epithelial cells and collapse of respiratory units in the gills, likely resulting in acute oxygen deficit of vulnerable organs like the brain and the heart. The muscle rigour sometimes reported in salmon suffering from SGPVD could also be due to hypoxia, although ion disturbances (calcium) and CNS affection may also play a role. However, the rapid, synchronised, fatal disease course (approx. 4–6 days) with orange (jaundice-like) skin discolouration and extensive erythrophagocytosis in spleen and kidney haematopoietic may indicate other disease mechanisms than hypoxia alone. Another condition of such high lethality is haemophagocytic lymphohistiocytosis (HLH) secondary to several aetiologies, including viral infections like pandemic influenza and coronaviruses. A vicious circle of non-protective systemic inflammation («cytokine storm») fuelled by an increasing infectious load leads to hemodynamic disturbances and multiple organ failure. Moreover, in secondary HLH, all types of blood cells are phagocytosed, while in SGPVD, only RBCs are phagocytosed, i.e., an erythrophagocytosis. While rapid erythrophagocytosis resembles HLH, the absence of multi-lineage phagocytosis and minimal inflammatory gene expression indicates a distinct mechanism. However, transcriptomics cannot rule out that preformed cytokines could be released. Therefore, as a classical cytokine storm is not evident in this study, parallels to HLH-like syndromes in mammals warrant further study. In addition, the complexity of inflammatory conditions and the rudimentary clinical diagnostics and biomarkers of fish make it difficult at present to resolve the disease mechanisms exacerbating SGPVD to mass mortality. The erythrophagocytosis of SGPV is unexplained and appears to have another cause other than in infectious salmon anaemia (ISA). Possibly the RBCs are damaged passing through the severely diseased gill and thus expose «eat me signals» leading to a high rate of phagocytosis by the system intended for this purpose in the spleen and kidney. Therefore, erythrophagocytosis likely indicates RBCs damage or clearance, which could be secondary to hypoxia or local vascular injury associated with severe gill pathology. Similarly, the up-regulation of coagulation genes may reflect a pro-thrombotic or inflammatory state, potentially linked to tissue damage or immune activation. Future studies could help to validate these associations and determine their predictive value for disease severity.

## Conclusion

In conclusion, SGPVD affects haematopoietic organs, leading to red blood cell breakdown and distinct immune responses. Gene expression patterns reveal immune activation, antiviral defence, coagulation, and stress-related pathways. These findings enhance our understanding of the disease’s systemic effects and support improved management strategies.

## Materials and methods

### Clinical observations and sampling

Atlantic salmon were sampled from a Norwegian salmon hatchery during an SGPVD outbreak as previously described [10]. To cover different stages of the disease course, fish were sampled from tanks with different disease statuses, and at three different time points during the outbreak (Fig. S1). Briefly, fish were collected at three different time points during the SGPVD outbreak and from three different tanks. First, fourteen Atlantic salmon with a mean weight of 30 g (tank I) with clinical signs of SGPVD were sampled at the onset of mortality (Table 1). On the same day, ten fish of the same size from a tank with apparently healthy fish (tank III) were sampled (Table 1). The next sampling, from eight fish from tank I, was performed after eight days when the disease outbreak had ceased (Table 1). Signs of SGPVD and mortality were then observed in a new tank (tank II), where eight fish were sampled two days after SGPVD onset (Table 1). Eight apparently healthy fish with an average weight of 41 g were sampled from tank III at the same time point, intended as negative controls. The kidney, spleen, heart, liver, pancreas with pyloric caeca, skin, and muscle, in addition to gills characterised earlier [10], were collected and kept in 10% neutral phosphate-buffered formalin. Kidney and spleen samples were stored in RNA*later*™ (Invitrogen™).

**Table 1:** A summary of kidney and spleen data from the natural outbreak of salmon gill poxvirus disease presented in this study. *Indicates the number of fish autopsied, sampled, and previously assessed for gill histopathology [10]. **Represents the number of fish analysed for histopathology and RNA-Seq of the spleen and kidney. These groups also include fish previously examined for gill histopathology and gene expression analysis [10]. A lower Ct value corresponds to a higher viral load.

Date of sampling	Tank ID	Group ID	Clinical state	Number of fish	SGPV Ct kidney Median (range)	SGPV Ct spleen Median (range)
May 10^th^	I	M_I	diseased	5** (14*)	34.2 (32.6–38.1)	33.3 (28.6–42.1)
May 18^th^	I	L_I	recovered	4** (8*)	0/4	0/4
May 23^rd^	II	M_II	diseased	5** (8*)	38.8 (34.2–42.0)	3.7 (30.7–41.2)
May 10^th^	III	C_III	healthy	6** (10*)	0/6	0/6
May 23^rd^	III	E_III	healthy	4** (8*)	0/4	0/4

### Histology, RNAscope, and qPCR for SGPV detection

Histology was performed using standard protocols. Section (3 μm-thick) were stained with haematoxylin and eosin (H&E), by MSB to investigate the staining properties of deposits in the spleen and kidney and localise fibrin [51], and by Prussian blue to assess the presence of ferric iron [52]. Deposits, suspected to originate from phagocytosed RBCs, exhibited a yellow colouration consistent with MSB staining, indicative of haemoglobin presence. RNAscope for SGPV was carried out to complement qPCR analyses [12, 13]. The kidney and spleen were tested for SGPV as previously described [10].

### RNA extraction and sequencing

To ensure consistency across tissue types and enable direct comparison of transcriptomic responses, we selected at least 4 biological replicates per organ, matching the previously published gill transcriptome analysis [10]. This design enhanced our ability to distinguish systemic from organ-specific responses to SGPV infection. The sample size was chosen based on the prior study [10] demonstrating sufficient power to detect biologically meaningful differences in gene expression while accounting for inter-individual variability. To address potential tank or batch effects, we implemented the randomisation of fish sampled from tanks with different disease statuses. Moreover, PCA was used to assess and visualise potential batch effects.

RNA was extracted from 24 kidney and spleen samples, respectively, with the MagNA Pure 96 Cellular RNA Large Volume Kit (Roche) and the MagNA Pure 96 Instrument (Roche). Sample quality was checked using the Thermo Scientific™ Multiskan Sky Microplate Spectrophotometer and the TapeStation 4150 Systems (Agilent), ensuring 260/280 > 2 and RIN > 8. Library preparation was performed with the TruSeq^®^ Stranded mRNA kit (Illumina) and sequenced with the S4 NovaSeq ¼ flow cell (300 cycles), resulting in approx. 2-2.5 billion reads per end.

### Bioinformatics analyses

For RNA-Seq data, raw reads were quality checked and pseudo-aligned against the Atlantic salmon reference transcriptome cDNA file in Ensembl v3.1 (last modified 2023-04-22) from the biomaRt v2.60.0 package [53, 54] using Kallisto v0.48.0 [55] in RStudio v2024.04.0 [56]. Transcript-level expression was imported into R v4.4.0 [57] and summarised to the gene level using the R/tximport v1.32.0 [58]. To account for library size differences, raw counts were transformed into log_2_-counts per million (CPM), then filtered and normalised to ensure that the expression distributions of each sample were similar across the entire experiment. Normalisation was performed by trimmed mean of M-values (TMM) [59] using the calcNormFactors function in edgeR v4.2.0 package [60] (Fig. S3A-C), followed by the limma v3.60.0 package [61] with its voom method, linear modelling and empirical Bayes moderation to assess the differential expression and perform gene set testing. Genes with log_2_ fold change of at least│1│ and Benjamini-Hochberg [62] false discovery rate adjusted p-value ≤ 0.05 were considered to be differentially expressed. Gene Ontology (GO) enrichment analyses were performed in R using Bioconductor packages limma, gprofiler2 v0.2.3 [63, 64], clusterProfiler v4.12.0 [65] and enrichplot v1.24.0 [66].

## Electronic supplementary material

Below is the link to the electronic supplementary material.

Supplementary Material 1

Supplementary Material 2

## Data Availability

All data needed to evaluate the conclusions in the paper are present in the paper and / or the supplementary materials. RNA-Seq data are available under SRA accession PRJNA1190207.

## Abbreviations

SGPVD: Salmon gill poxvirus disease
SGPV: Salmon gill poxvirus
TUNEL: Terminal deoxynucleotidyl transferase dUTP nick end labelling
RBCs: Red blood cells
MX: Myxovirus resistance
IFNγ: Interferon-gamma
GRAA: Granzyme A
MSB: Martius, Scarlet and Blue
PCA: Principal component analysis
DEGs: Differentially expressed genes
SOCS: Suppressor of cytokine signalling
TNFL6: Tumour necrosis factor ligand superfamily member 6
HMOX1a: Heme oxygenase 1a
AICD: Activation-induced cell death
ANGL4: Angiopoietin-related protein 4
CXL10: C-X-C motif chemokine 10
C209E: C-type lectin domain containing 1
IFNGR1l1: Interferon-gamma receptor 1-like 1
IGF1: Insulin-like growth factor 1
CD8a: Cluster of differentiation 8a
CCR10: Chemokine (C-C motif) receptor 10
EPOR: Erythropoietin receptor
DMTN: Dematin actin-binding protein
HDR: Haematopoietic death receptor
PD: Pancreas disease
ISAV: Infectious salmon anaemia virus
VHSV: Viral haemorrhagic septicaemia virus
IHNV: Infectious haematopoietic necrosis virus
HLH: Hemophagocytic lymphohistiocytosis
ISA: Infectious salmon anaemia
H&E: Haematoxylin and eosin
CPM: Counts per million
TMM: Trimmed mean of M-values
