# Supplementary material for: Severe pathological and transcriptional changes in haematopoietic organs of salmon suffering from salmon gill poxvirus disease
Source: BMC Vet Res. 2025 Jul 16;21:471. doi: 10.1186/s12917-025-04922-6 (PMC12265247; doi:10.1186/s12917-025-04922-6)
Supplement: Supplementary file 2 — Supplementary Material 2 [file 12917_2025_4922_MOESM2_ESM.docx]

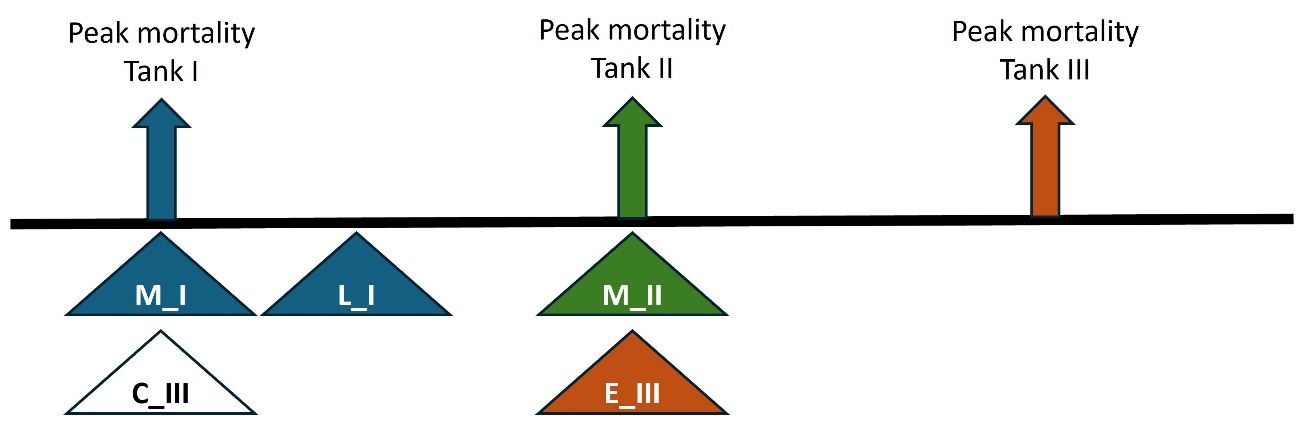


**Fig. S1. Summarized schematic view of the sampling and experimental design timeline.**


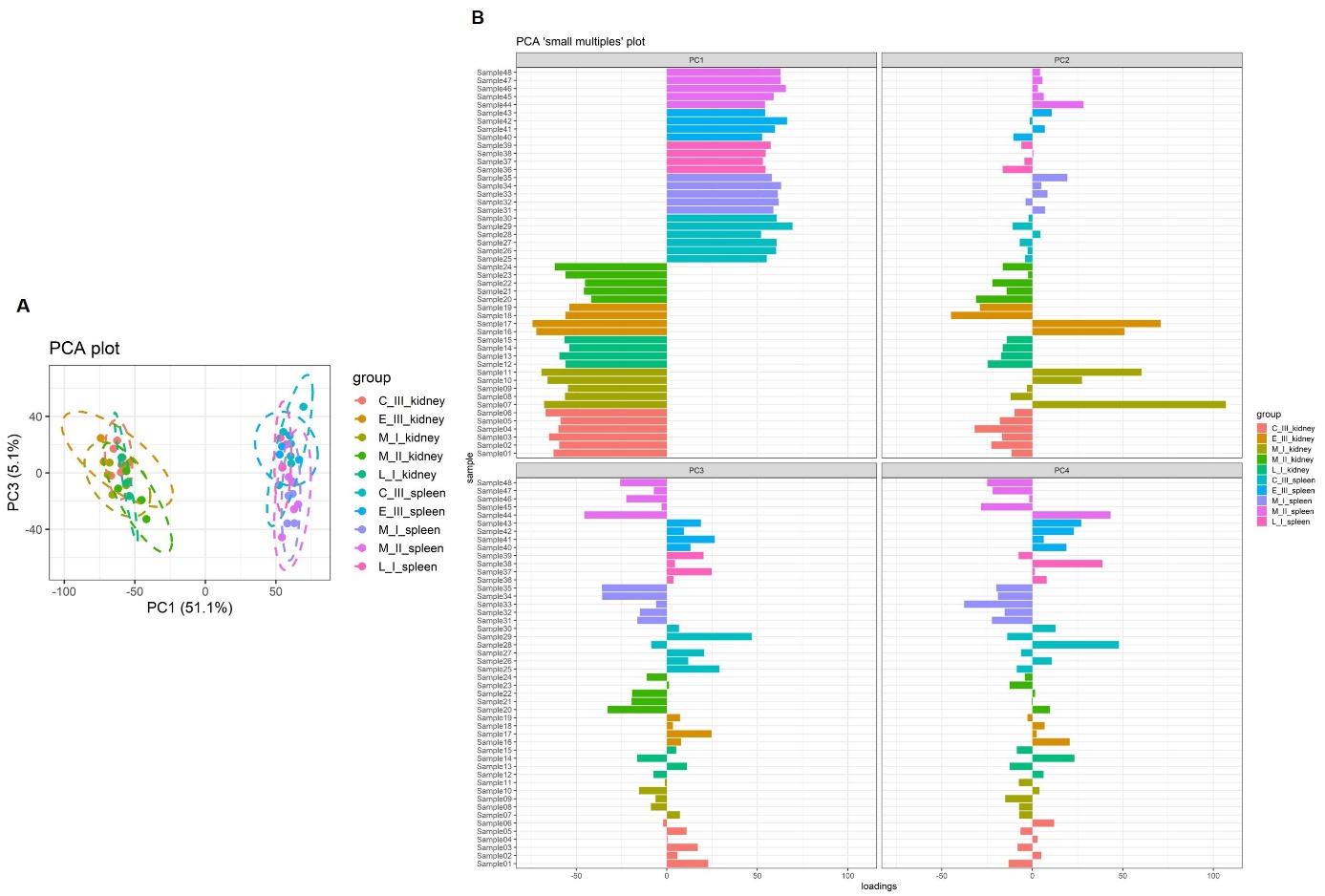


**Fig. S2. Principal component analysis (PCA) allows to summarize and to visualize the information in a data set containing individuals/observations described by multiple inter-correlated quantitative variables.** **A)** PCA showing the clustering of all RNA-Seq data. **B)** PCA multiple small barplots to show which variables have high loading and in which principal components.


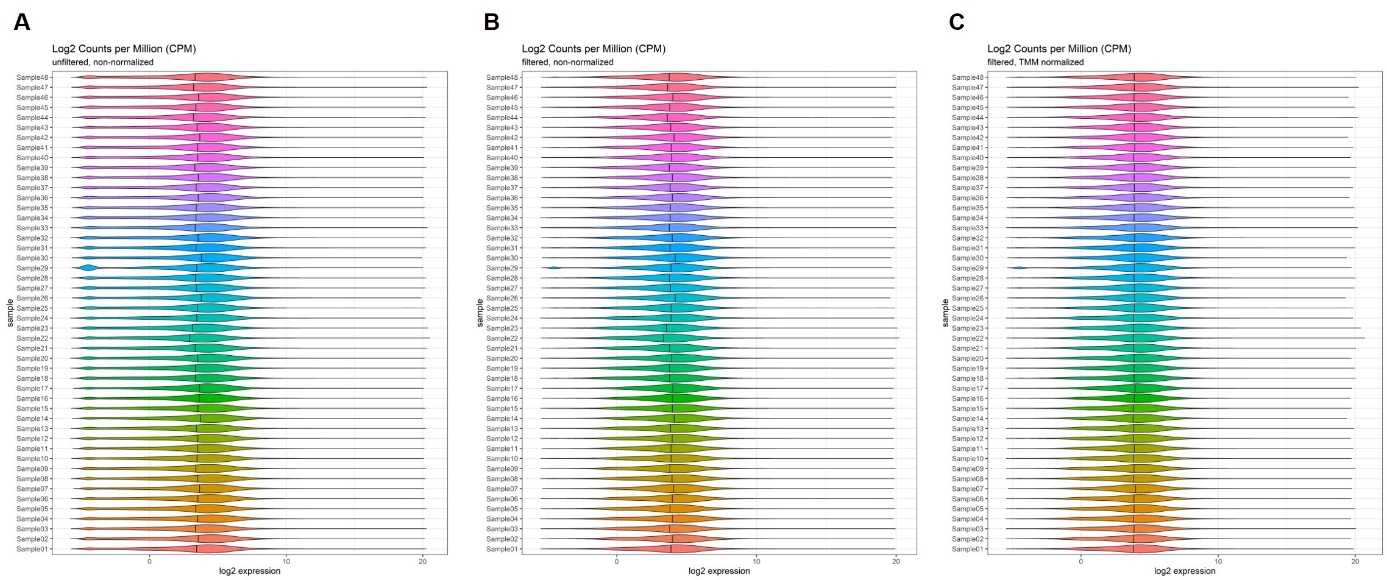


**Fig. S3.** **Transformation of raw counts onto a scale that accounts for library size differences.** **A)** For the transformation, it was used the log2-counts per million (CPM) as it does not account for gene length differences. **B)** Genes that do not have a worthwhile number of reads in any sample were filtered out. **C)** Normalisation is required to ensure that the expression distributions of each sample are similar across the entire experiment. Normalisation was performed by the method of trimmed mean of M-values (TMM) [1] using the calcNormFactors function in edgeR [2].

**References:**

1. Robinson MD, Oshlack A: **A scaling normalization method for differential expression analysis of RNA-seq data**. *Genome Biol* 2010, **11**:1-9.

2. Robinson MD, McCarthy DJ, Smyth GK: **edgeR: a Bioconductor package for differential expression analysis of digital gene expression data**. *Bioinformatics* 2010, **26**(1):139-140.
